# Coexistence of Gait Disturbances and Chorea in Experimental Huntington's Disease

**DOI:** 10.1155/2015/970204

**Published:** 2015-05-06

**Authors:** João Casaca-Carreira, Yasin Temel, Marloes van Zelst, Ali Jahanshahi

**Affiliations:** ^1^Department of Neuroscience, Maastricht University Medical Center, 6229 ER Maastricht, Netherlands; ^2^European Graduate School of Neuroscience (EURON), 6229 ER Maastricht, Netherlands; ^3^Department of Neurosurgery, Maastricht University Medical Center, 6229 ER Maastricht, Netherlands

## Abstract

Huntington's disease (HD) is an autosomal dominant neurodegenerative disease caused by an expanded CAG repeat. The clinical features are progressive motor dysfunction, cognitive deterioration, and psychiatric disturbances. Unpredictable choreic movements, among the most characteristic hallmarks, may contribute to gait disturbances and loss of balance in HD individuals. In this study, we aimed to investigate and characterize the gait abnormalities and choreic movements in a transgenic rat model of HD (tgHD). TgHD presents typical neuropathological, neurophysiological, and behavioral aspects mimicking some of the key features of human HD and is the only described experimental model for HD that exhibits choreiform movements. We used the Catwalk, with emphasis on static and dynamic gait parameters, to test the hypothesis that at symptomatic age (9 months) the dynamic measures of gait in HD are altered and coexist with choreiform movements. Our results showed that the dynamic parameters seem to be more affected than static parameters at this age in tgHD rats. The number of steps and step cycles and swing speed of the paws were increased in tgHD rat in comparison to wild-type controls. Our study demonstrates that gait abnormalities coexist with chorea rather than being caused by it. These symptoms may originate from distinct networks in the basal ganglia and downstream connections.

## 1. Introduction

Huntington's disease (HD) is an autosomal dominant neurodegenerative disease caused by an expanded CAG repeat on the short arm of chromosome 4 [1]. The clinical features are progressive motor dysfunction, cognitive deterioration, and psychiatric disturbances that begin around midlife [2]. However, subtle functional deficits occur years prior to clinical diagnosis [2]. In the early stages of the disease, gait may become unstable [3]. Moreover, slight motor abnormalities, like finger tapping, tongue protrusion, and tandem gait, in premanifest HD (gene carriers not yet demonstrating motor symptoms and functional decline), are associated with an early onset of the disease and smaller striatal volumes [4].

The motor symptoms are among the most characteristic hallmarks, which makes chorea a defining symptom of the disease, known as Chorea or Huntington's chorea. Greek word for dance, chorea, is defined as an unpredictable, rapid, involuntary, nonrepetitive movement affecting the face, trunk, and/or limbs [5]. Besides, gait is another aspect of motor function that shows remarkable abnormalities in HD individuals, ranging from lateral or side-to-side deviations and imbalance to difficulties with turning and initiation of gait in some cases. Shorter step length, decreased walking speed, and increased stride time have also been reported in HD patients [6–8]. It has been hypothesized that choreic movements cause gait disturbances and loss of balance in HD individuals [9]. For instance, the augmented gait such as stride-to-stride variability observed in HD is suggested to be due to the excessive choreic movements [6, 8, 10]. In addition, chorea appears to influence factors that contribute to falls in HD. Particularly, clinical scores of chorea were positively associated with the range of angular trunk motion in both mediolateral and anterioposterior directions in HD individuals [3]. Nonetheless, gait and balance abnormalities do not seem to be purely caused by chorea, regarding the fact that distinct neural networks are shown to underlie these symptoms [11–16]. For instance, clinical data suggest that dysfunction of the basal ganglia cueing mechanism results in impaired locomotion timing in HD. This was illustrated by difficulties in ability to regulate rhythm, an increased variability of stepping rates, and difficulties in achieving synchronized footsteps [8].

To address whether gait is “physically” affected by chorea in HD, we investigated gait and chorea in quadrupedal animal, where balance can be independently accessed without being affected by other symptoms such as chorea.

The discovery of the HD gene in 1993 [1] facilitated the development of several genetically modified rodent models that mimic the onset and progression of the disease. Among those, transgenic rat model of HD (tgHD) has widely been used [17] and presents typical neuropathological, neurophysiological, and behavioral features resembling the human condition [18, 19]. The tgHD is the only described experimental model of HD exhibiting choreiform movements [9, 17, 18, 20]. The onset of hyperkinetic movements is at the ages of 6-8 months, which might differ from one colony to another, and its severity increases with disease progression [9]. In tgHD rats gait abnormalities have been assessed in presymptomatic stages (2, 3, and 4 months of age) showing a hyperkinetic profile with increased swing and locomotor speed and decreased paw contact [21]. Following the presymptomatic phase of the disease, hyperkinetic profile changes to hypokinesia in the symptomatic/late phase [17–19, 21]. In this study, we analyzed the static and dynamic parameters during the early symptomatic phase of the disease in tgHD rats. As it was reported before [21], the dynamic parameters tend to be more affected in the early phase of the disease. Here, we decided to analyze also the static parameters, since these parameters are likely to be affected at later ages when the hypokinetic profile of the disease begins to appear.

Automated and quantitative gait analysis method Catwalk [22] was initially used in animal models of spinal cord injury. To date, it is a well-established method to assess gait abnormalities in several models of neurological disorders [21, 23, 24]. The Catwalk provides both dynamics, for example, inter- and intralimb coordination, weight bearing, duration of swing, and stance phases and static parameters of gait, like print length, stride length, base of support, and so forth, in an objective and efficient approach. We used the Catwalk, accessing the static and dynamic gait parameters, to test the hypothesis that gait abnormalities coexist with choreiform movements in HD rather than being caused by it.

## 2. Methods

### 2.1. Subjects

TgHD rats (Sprague-Dawley background) express a 1962-base-pares rat HD cDNA fragment carrying a 51-CAG repeat under the control of the endogenous rat huntingtin promoter [17]. Homozygous male tgHD rats and wild-type (WT) littermates were housed in pairs under a 12 h reversed day/night cycle (light from 7 am till 7 pm), provided with water and food* ad libitum*. Room temperature (21 ± 2°C), humidity (60 ± 10%), and air exchange (16 times per hour) were automatically controlled. The Animal Experiments and Ethics Committee of Maastricht had approved all experimental procedures.

### 2.2. Experimental Groups

WT (*n* = 7) and tgHD (*n* = 7) male rats were bred at the Central Animal Facility of Maastricht University (Maastricht, Netherlands). For genotyping purposes, all the animals were tail tipped at weaning and genotypes determined by PCR.

### 2.3. Behavioral Testing

All the behavioral tests were performed at the age of approximately 9 months and during the dark phase.

#### 2.3.1. Choreiform Movements

The number of choreiform movements was quantified using the same criteria as described previously [9, 20]. In brief, all the subjects were videotaped individually in a home cage, with sawdust from their own home cage. The animals had 15 minutes to acclimatize to the cage and room, before the videotaping started. The video recordings were all performed on the same day, during the dark phase and under infrared light. Two independent observers (blinded to the experimental groups) scored the total number of choreiform movements (abrupt, rapid, brief, and unsustained irregular movements of the neck) during an observation period of 20 min. Choreiform movements were not detected in other parts of the body.

#### 2.3.2. Automated Gait Analysis

**Figure 1:**
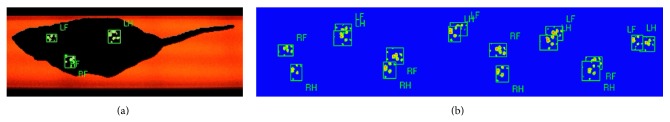
Screen shots represent the Catwalk gait analysis method. (a) A rat, running on the platform. (b) Representation of paw prints in the analysis software. RF: right front paw; LF: left front paw; RH: right hind paw; LH: left hind paw.

The gait analysis system Catwalk 7.1 (Noldus IT, Netherlands) consists of an enclosed walkway with a glass plate and a speed video recording camera (Figure 1). Gait performance was assessed and recorded using the Catwalk analysis software. The glass plate was cleaned and dried before testing each subject to minimize the transmission of olfactory clues and prevent animals from stopping to smell or explore something during a run.

One week prior to the first test session, rats were trained to walk down the runway daily [24]. The training and testing were done by a researcher, which was blinded to the genotype of the animals. As experienced elsewhere [21, 24], some of the animals showed reduced mobility or motivation to cross the complete runway within an acceptable timeframe. To overcome this, some strategies have been used, like prior acclimatization [21] or reducing the number of consecutive runways to include in the analysis [24]. In our case, we decided to test the animals until 3 complete runs were achieved. An acceptable run was defined as an uninterrupted walk of the runway. For every parameter, the average of three runs was used for the analysis.

The Catwalk analysis software produces several parameters, and in previous studies the criteria for the selection of these parameters are not always justified. Here, the decision to include these parameters was based on the only study using the Catwalk system in tgHD rats and also on human studies that performed gait analysis (Table 1).

### 2.4. Statistical Analysis

Individual runs were analyzed by the Catwalk software. Three individual runs from each animal were used to compute mean and standard error of the mean (SEM) for all the Catwalk parameters. A separate analysis was done for every parameter belonging to each paw, unless stated otherwise. Statistical analysis between the 2 experimental groups was done by applying independent sample *t*-test. Interobserver agreement on the animals that showed choreiform movements was assessed using the weighed kappa coefficient. Data are presented as means and standard error of means (SEM). All statistical analyses were performed with SPSS 20.0 version for Windows, with *P* values smaller than 0.05 considered significant.

## 3. Results

**Figure 2:**
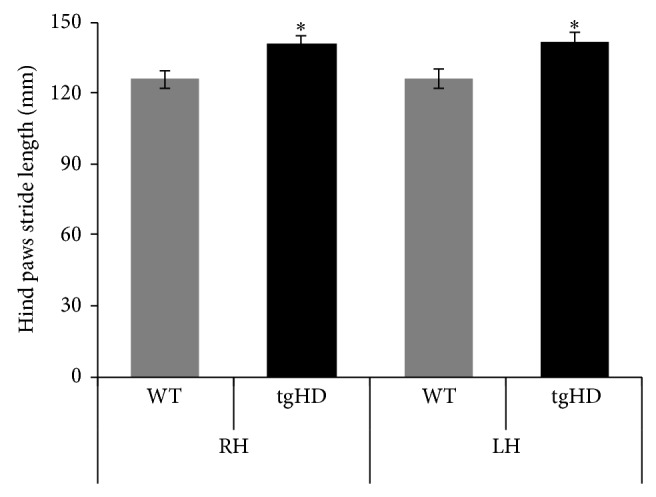
Static gait parameter—the graph represents the hind paws stride length in 9-month-old transgenic (tgHD) and wild-type (WT) rats. RH: right hind paw; LH: left hind paw. The values are mean ± standard error of the mean (SEM) and statistically significant difference (*P* < 0.05) is indicated by an (∗).

Among the static parameters analyzed, stride length (Figure 2) was augmented in tgHD rats (*P* = 0.02), while the base of support did not show any significant difference between groups (data are not shown).

**Figure 3:**
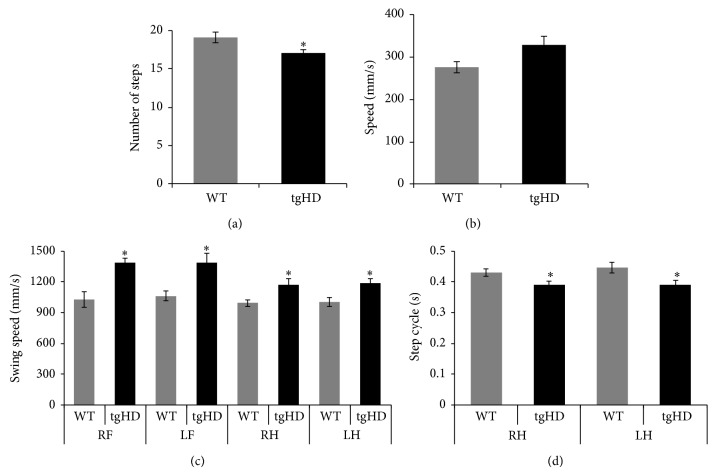
Dynamic gait parameters—(a) the graph shows the average number of steps, comparing transgenic (tgHD) with wild-type (WT) rats. (b) The graph represents the average speed in mm/s of the 9-month-old tgHD versus WT rats. (c) The graph shows the swing speed belongs to 4 paws of tgHD compared with WT rats. (d) The graph shows the step cycles in seconds for the hind paws from the tgHD compared with WT rats. RF: right front paw; LF: left front paw; RH: right hind paw; LH: left hind paw. The values are presented as mean ± standard error of the mean (SEM) and statistically significant difference (*P* < 0.05) is indicated by an (∗).

**Figure 4:**
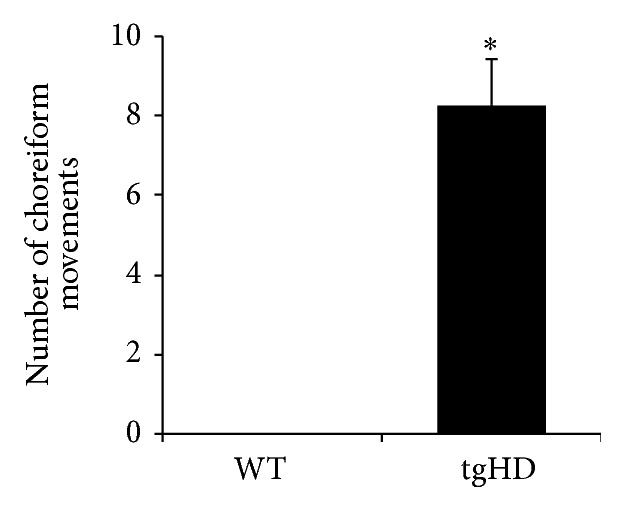
The graph represents the number of choreiform movements scored during 20 minutes. The values are presented as mean ± standard error of the mean (SEM) and statistically significant difference (*P* < 0.05) is indicated by an (∗).

In the dynamic parameters, tgHD rats showed decreased number of steps (*P* = 0.033) and a trend of increased speed (*P* = 0.051) when compared with age matched WT animals (Figures 3(a) and 3(b)). In addition, tgHD rats showed a higher swing speed in all paws (Figure 3(c), *P* = 0.002) and reduced hind paws step cycles (*P* = 0.033) in comparison with age matched WT subjects (Figure 3(d)). The number of choreiform movements was assessed in both groups at 9 months of age (Figure 4). The WT rats did not show any abnormal movements corresponding to the definition of choreiform movements described in this model. However, on average, the tgHD animals showed 8-9 movements during 20 min of observation.

Of note, kappa value was 0.857, interpreted as a very good interobserver agreement.

## 4. Discussion

Our behavioral study demonstrated that tgHD rats show gait abnormalities in the early symptomatic phase of the disease. Moreover, the manifestation of locomotor deficits is accompanied by choreiform movements, which are part of the hyperkinetic features of HD. In the adult form of HD, motor disturbances frequently involve hyperkinesia, chorea, and progressive akinetic/rigid symptoms at more advanced stages [7].

Among the static parameters of gait, base of support, and stride length, only the latter turned out to be significantly increased in the hind paws of the tgHD rats. These results are in line with earlier human studies, showing that stride length is frequently increased in HD subjects when compared to healthy controls [7]. Interestingly, reduction in stride length, which is considered as a hypokinetic feature, can be found at later ages, showing that gait follows the hyperkinetic and hypokinetic course of the disease in HD [7]. Of note, the phenotype of the tgHD rats comprises an early symptomatic phase, characterized by hypermobility and a late disease phase, where hypokinesia is the dominant symptom [18, 19, 25], resembling the human condition.

The fact that the base of support did not differ between the two groups can be explained by the age, at which the rats were evaluated. Due to the known hyperkinetic/hypokinetic profile of this model, we expect hypokinesia to appear later in the progression of the disease's late symptomatic phase. Base of support is by definition the average width between the front and hind paws and is frequently increased to compensate for an instable gait [26] and to increase balance [27], which we expect to occur at later stages of the disease in tgHD rats.

As we hypothesized, the dynamic parameters seem to be more affected at this age than the static parameters in tgHD rats. Our results showed that the number of steps, step cycles and swing speed were increased. All these parameters seem to indicate a hyperkinetic profile in tgHD rats, which is still detectable at 9 months of age. Dynamic parameters can be influenced by velocity [27]; yet in our study this seems to not be the case, since velocity was not statistically different between two groups. Nevertheless, there was a clear trend (*P* = 0.051) on the velocity parameter, where the tgHD group was faster than the wild-types. This trend might be interpreted as a sign that the hyperkinetic presymptomatic trait of this model [17–19] is becoming less apparent and the hypokinesia is starting to be the dominant symptom.

Through gait analysis, it was shown that tgHD rats in pre-manifestation phase have a hyperkinetic profile, characterized by increased swing speed, decreased paw contact, and increased speed [21]. This hyperkinetic profile was not detectable on the Rotarod test but only with the Catwalk, which seems to be a more sensitive method for detecting locomotor deficits. Notably, 2- to 5-month-old rats at premanifest stage of HD were examined in this study, but only at 2 months of age an increased speed was detected. According to previous reports [9, 17–20], at these “early” ages the tgHD do not fully express the disease profile, while 9-month-old rats were used in our study, which seems to be the onset time of the neurodegeneration phase. Besides establishing the gait assessment, we also replicated the presence of choreiform movements, which so far have been only identified in this rodent model. Such feature makes tgHD a suitable model to study these particular motor symptoms and possible relations with other motor symptoms. On average, we observed 8-9 choreiform movements of the tgHD rats in a time frame of 20 minutes. The number of choreiform movements that was scored in our experiment was substantially lower than those described earlier [9, 20]. This might be explained by the age of the animals tested, since the number of choreiform movements tends to increase with age. In our study, the animals were assessed at 9 months of age, the early symptomatic phase, while previously the animals had been assessed in their late symptomatic phase [9, 20].

Our results appear to be in line with the human studies, which show that chorea has an influence on gait. However, a more detailed interpretation of the number of choreiform movements observed would not be able to predict a direct effect of chorea on gait. In fact, the probability of overlapping a choreiform movement with the duration of the Catwalk run seems to be unlikely. A direct relation between chorea and gait deficits, decreased balance, and falls has been suggested before [3, 7], whilst others have questioned this relation. In a clinical study, Reynolds and coworkers [10] investigated the gait in 6 HD patients and found out that chorea in HD does not considerably affect the center of gravity during ambulation, despite random and frequent variability during gait cycle. An earlier study [6] reported that, even when chorea was clearly improved with neuroleptics, gait abnormalities were still detectable, mainly variability in swing and stance times. Moreover, in favor of this reasoning is the fact that gait abnormalities were detected at 2 months of age in tgHD [21] when choreiform movements are not yet present. Another argument supporting the hypothesis that gait deficits are not influenced by chorea arises from the quadruped locomotion of the rats, which is traduced in a more stable and wide base of support. Consequently a movement of the head—choreiform movement—would not be enough to provoke an alteration on the number of steps and step cycles and swing speed, as we have demonstrated.

This apparent contradiction hints once more the coexistence of hyperkinetic and hypokinetic disturbances in HD, possibly due to the involvement of different mechanisms/pathways in the pathology of motor symptoms. Our previous works have shown that (i) the tgHD rats exert a hyperdopaminergic status, expressed in an increased number of dopaminergic cells in the brainstem, accompanied by an enhanced dopamine expression in the striatum [28, 29]; (ii) the choreiform movements can be reduced by the administration of tetrabenazine, a reversible inhibitor of the vesicular monoamine transporter [9], similar to clinical practice. Based on these findings, it can be suggested that the choreiform movements are originated due to hyperactivity of the nigrostriatal dopaminergic system.

On the other hand, gait changes identified in HD can be linked to deficits in striatum, namely, caudate and putamen atrophy [30]. Sequential and preferential loss of different types of striatal projection neurons in HD may explain distinct origins for chorea and gait [11–16]. According to human postmortem studies, the early loss of GABAergic striato-substantia nigra pars reticulata neurons may cause the hyperactivity of dopaminergic neurons leading to early appearance of chorea. In addition, the loss of striato-globus pallidus externus neurons, which suppress undesirable movements, takes place earlier than striato-globus pallidus internus neurons, which control and promote wanted movements. This suggests that the appearance of akinesia and, consequently, the gait abnormalities have a different anatomical substrate.

In conclusion, we suggest that gait abnormalities in tgHD rats originate from the striatal deficits and possibly impairment of the executive functions. On the other hand, chorea is seemingly caused by dysregulation of the nigrostriatal dopaminergic pathway.

## Figures and Tables

**Table 1:** Description of the parameters selected and analyzed from the Catwalk test and respective units of measure.

Parameter	Description	Unit
Base of support (BOS)	Average width between front and hind paws	mm

Stride length	Distance between successive placements of the same paw	mm

Step cycle	Time between 2 consecutive contacts with the glass plate of the same paw (stance + swing phase)	s

Swing speed	Speed of the paw during the swing phase	mm/s

Average speed	Distance covered divided by the time used to cross	mm/s

Cadence	Number of steps per second	

Number of steps	Number of steps during a run	
